# Polarization-based smoke removal method for surgical images

**DOI:** 10.1364/BOE.451517

**Published:** 2022-03-22

**Authors:** Daqian Wang, Ji Qi, Baoru Huang, Elizabeth Noble, Danail Stoyanov, Jun Gao, Daniel S. Elson

**Affiliations:** 1 School of Computer and Information, Hefei University of Technology, Hefei, 230601, China; 2 Hamlyn Centre for Robotic Surgery, Imperial College London, London, SW7 2AZ, UK; 3 Department of Surgery and Cancer, Imperial College London, London, SW7 2AZ, UK; 4 Research Center for Intelligent Sensing, Zhejiang Lab, Hangzhou, 311100, China; 5 Department of Computer Science, University College London, London, WC1E 6BT, UK; 6 dw619@mail.hfut.edu.cn; 7 ds.elson@imperial.ac.uk

## Abstract

Smoke generated during surgery affects tissue visibility and degrades image quality, affecting surgical decisions and limiting further image processing and analysis. Polarization is a fundamental property of light and polarization-resolved imaging has been studied and applied to general visibility restoration scenarios such as for smog or mist removal or in underwater environments. However, there is no related research or application for surgical smoke removal. Due to differences between surgical smoke and general haze scenarios, we propose an alternative imaging degradation model by redefining the form of the transmission parameters. The analysis of the propagation of polarized light interacting with the mixed medium of smoke and tissue is proposed to realize polarization-based smoke removal (visibility restoration). Theoretical analysis and observation of experimental data shows that the cross-polarized channel data generated by multiple scattering is less affected by smoke compared to the co-polarized channel. The polarization difference calculation for different color channels can estimate the model transmission parameters and reconstruct the image with restored visibility. Qualitative and quantitative comparison with alternative methods show that the polarization-based image smoke-removal method can effectively reduce the degradation of biomedical images caused by surgical smoke and partially restore the original degree of polarization of the samples.

## 1. Introduction

In surgery, the production of surgical smoke, vapor and aerosols (referred to throughout as ‘smoke’) during tissue dissection not only reduces the tissue visibility, but also introduces an error during further image processing and tissue diagnosis [1]. Therefore, it would be useful to reduce the influence of surgical smoke, enhance image contrast and texture, and remove ‘noise’ in order to improve the biomedical image quality.

Polarization is a fundamental property applying to transverse waves that specifies the geometrical orientation of the oscillations. According to the oscillation trajectory of the electric field in the plane perpendicular to the propagation direction, polarized light can be divided into different polarization states, namely linearly polarized light, circularly polarized light and elliptically polarized light. Polarized light can be produced from the common physical processes that deviate light beams, including scattering, absorption, refraction, reflection and birefringence. When light propagates through a scattering medium, it undergoes multiple scattering with particles, leading to randomization of its direction, phase, and polarization state compared with the incident light. Therefore, compared to general non-polarized optical imaging, polarization imaging can detect additional or ‘hidden’ information from the way the scattering causes variation in the polarization state of light. On this basis, polarization has been widely applied in many different fields, including for biomedical imaging and tissue diagnosis [2–5].

Although the problem of surgical smoke removal is a recent topic of interest with only a few published studies [6–10], it builds on the basis of the well-established outdoor image dehazing research. Outdoor dehazing methods can be roughly divided into three main categories: enhancement approaches, restoration approaches and learning approaches [11]. Enhancement approaches seek to remove the haze (‘noise’) and improve the contrast by using image processing algorithms including, for example, adaptive histogram equalization (AHE) [12], retinex [13], wavelets [14], etc. These methods are simple and direct but their dehazing effect is limited. Restoration approaches are usually based on various models that estimate the dehazed image color and intensity from the image based on specific priors or assumptions, for example, based on the dark channel prior [15], maximizing contrast [16], color lines prior [17], haze lines prior [18,19], color attenuation prior [20] and polarization based [21–25], etc. These methods usually have a better dehazed output, but the assumptions and prior information have limitations and are not applicable in all scenarios. Learning approaches are recently becoming popular, some of which still follow the idea of estimating model parameters, while others propose a trainable end-to-end network for direct haze-free output [26,27]. However, learning approaches need advanced equipment, specific datasets and a lot of training data and time.

Even though there are examples of polarization imaging in outdoor scenes such as for foggy days [21], underwater imaging [22–25], and for biomedical image processing and tissue diagnosis [2–5], there are currently neither related research about polarization based surgical smoke removal, nor polarized surgical smoke datasets [1]. In this paper, we propose a polarization-based smoke removal method for biomedical images. We analyzed and simulated the propagation of polarized light in a mixed medium, and implemented the polarization difference method to estimate the transmission parameters of the smoke. Real experiments were performed to verify the proposed method, and qualitative and quantitative results show that our method effectively reduced the degradation of biomedical images caused by surgical smoke and partially restored the original degree of polarization of the samples. The proposed method has advantages of simplicity, speed and effectiveness, without a requirement for extensive datasets or training process. Medical images can be restored from the effect of surgical smoke in real time.

The remainder of this paper is organized as follows. In Sec. 2, the general image degradation model is introduced and a redefined polarization-based image degradation model is proposed. In Sec. 3, the propagation of polarized light is analyzed and simulated and the detailed process of parameter estimation from smoke data is proposed, including the polarization difference calculation for the estimation of transmission. Then, in Sec. 4, the experimental results and qualitative and quantitative analysis are presented and finally some conclusions are drawn in Sec. 5.

## 2. Model and simulation

### 2.1 General image degradation model

In large-scale scattering media such as atmospheric and underwater environments, a common general outdoor image degradation model is [15]:

(1) I(x)=J(x)t(x)+A(∞)(1−t(x)),

where the two terms represent the direct transmission and scattered air-light, respectively. *I* and *J* describe the intensity of the haze and haze-free image, respectively, x is a pixel in the image, A(∞) denotes the atmospheric radiance at an infinite distance that scatters into the imaging path, *t* is the medium transmission rate which describes the proportion of radiance attenuation. The transmission rate *t* can be expressed as:

(2) $$t\text{(x)}=e^{-\beta d(x)},$$

when the medium extinction coefficient β is depth invariant and *d* refers to the scene depth.

### 2.2 Polarization-based Image degradation model

The image degradation model introduced above is applicable to a wide range of media where the estimated transmission map is related to the scene depth and haze density does not vary extensively across the scene. However, in a local, limited depth range, some definitions of the parameters of the degradation model should be modified. Depth information is independent from the attenuation of signal transmission *t*, because the depth of the local area is almost the same. Instead, the transmission rate *t* is determined by the optical depth of the local smoke τ , i.e. the higher the concentration of smoke, the lower the transmission rate. Therefore, we redefine the transmission *t* as:

(3) $$t\text{(x)}=e^{-\beta \tau (x)},$$

Correspondingly, A(∞) denotes the intensity of ambient light with infinite optical depth, meaning that the smoke is heavy enough that no object radiance can be received through the smoke. Therefore, the polarization-based smoke removal model in a specific polarization state can be rewritten as:

(4) $$I_{pol}\text{(x)}=J_{pol}\text{(x)}e^{-\beta \tau (x)}+{\text{A}}_{pol}(\infty )(1-e^{-\beta \tau (x)}),$$

### 2.3 Propagation of polarized light in a medium

Before developing solutions to estimate parameters of the degradation model and remove the smoke, it is important to understand how the polarized light interacts with the biological object and the smoke. In general, for surgical imaging the illumination must travel through the smoke layer, with photons then scattered by the object being imaged in reflection mode. As the light propagates into the scattering medium, its direction, phase, and polarization state become randomized due to multiple scattering [28,29]. The characteristics of the resulting scattered polarized light strongly depend on different parameters, such as the medium particle size and density, the initial polarization state, the wavelength, and the depth, among others [29,30].

**Fig. 1.**
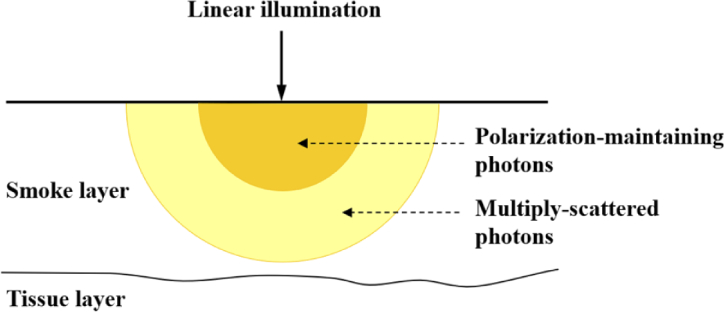
Schematic of polarized light propagation in the medium.

Ideally, the scene would be simplified as a double layered scattering medium, with an upper layer of smoke and a deeper layer of tissue, as shown in Fig. 1. For linear polarization illumination, the superficial smoke layer, as indicated in the orange semicircle area in Fig. 1, maintains the initial polarization, which means the backscattered polarized light contains a substantial component in its original polarization state, while the deeper layers contain a multiply-scattered component [28,31]. In order to study the propagation of polarized light in the medium under different illumination and medium conditions, simulation experiments were completed with a Polarized Monte Carlo program [32,33]. Polarized Monte Carlo provides a solution for understanding the propagation of polarized photons in the medium. It can sequentially track the movement of photons in the medium, and can realize the update of the photon’s polarization state after each movement. The detailed Polarized Monte Carlo modeling method can be found in the supplemental document. As surgical smoke consists of particles with diameters mostly in the range from 0.01 µm to 6.0 µm, depending on the type of electrocauterized tissue and surgical tool [34,35], we chose two particle diameters, 0.2 µm and 6.0 µm. The scattering coefficient (expressed as a number proportional to the amount of photons scattered per distance) is a measure of the ability of particles to scatter photons out of a beam of light and it is determined by the particle diameters, medium density, medium relative refractive index and incident wavelength [36]. The density was set to ensure that the calculated scattering coefficient was consistent with real smoke [37] and the absorption coefficient was set to a small value [38,39]. The scattering coefficient and anisotropy *g* were calculated by calling a Mie function. The refractive indexes of smoke particle and tissue were selected according to [40,41]. The detailed simulation parameters are listed in Table 1.

**Table 1.** Simulation parameters

Parameters	0.2 µm	6.0 µm
Illumination	Horizontal Linear Polarized	Horizontal Linear Polarized
Detection	Co-polarized and Cross-Polarized	Co-polarized and Cross-Polarized
Wavelength	0.630 µm	0.630 µm
Density [37]	$2\times 10^{\text{ - }3}\text{particles}/\mu {\text{m}}^{3}$	$\text{1}\times 10^{\text{ - 6}}\text{particles}/\mu {\text{m}}^{3}$
Anisotropy *g*	0.2052	0.7324
Scattering coefficient	0.1737cm^−1^	0.6230 cm^−1^
Absorption coefficient [38,39]	0.0100 cm^−1^	0.0100 cm^−1^
Refractive index (Smoke particle) [40]	1.57 + 0.4277i	1.57 + 0.4277i
Refractive index (Tissue) [41]	1.50	1.50
Number of photons	50000	50000

**Fig. 2.**
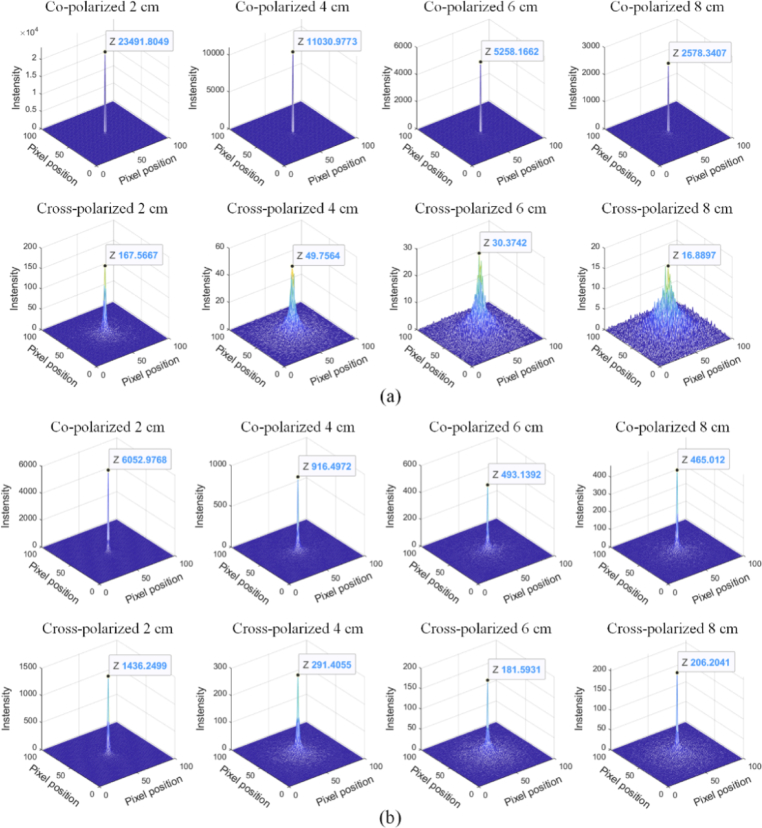
Radiance of polarization channels at different heights in the medium with (a) small-sized particles (0.2 µm) and (b) large-sized particles (6.0 µm). The radiance of the brightest central region is marked in each subgraph.

In the Monte Carlo calculations, the receiving plane was placed at different heights above the tissue – corresponding to different medium depths – to capture the backscattered radiance. The receiving plane had a size of 25×25 cm, with 100×100 sampling points, and the pencil beam illuminant was incident perpendicularly at the central point of the plane. The 3D distributions of linear polarized backscattered radiance in different types of medium at different heights, 2 cm, 4 cm, 6 cm and 8 cm, are shown in Fig. 2. For two media with particles of different sizes, regardless of medium depth, the co-polarized component dominated the backscattering radiance but decreased rapidly with increasing medium depth. Specifically, due to the small scattering coefficient of the small-sized particles, the probability of photon-particle interactions was low, and most photons were detected after being reflected directly by the object. Therefore, the backscattered co-polarized radiance was high and the cross-polarized radiance remained low. The scattering coefficient and the probability of photons-particle interactions increased for large-sized particles, and photons gradually lost their initial polarization state and became randomly polarized. The difference in radiance between co-polarized and cross-polarized channels decreased, especially when the depth of the medium exceeded the mean free path of photon transmission.

**Fig. 3.**
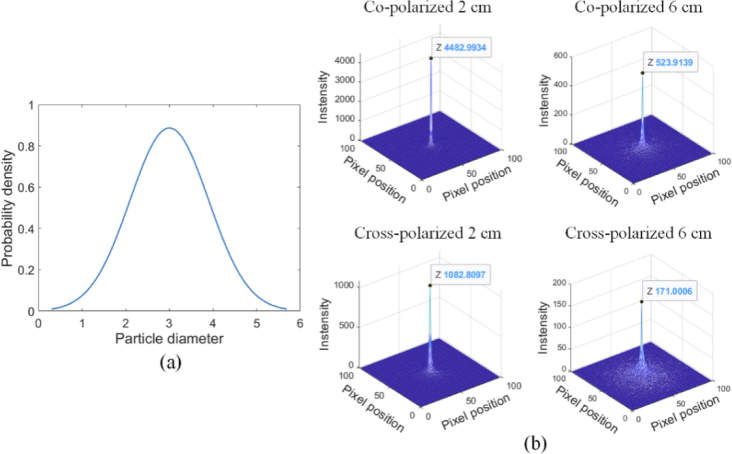
Backscattered radiance of linear polarization illumination in a polydisperse medium: (a) the distribution of the particle sizes and the radiance of (b) co-polarized and cross-polarized channel at different heights.

Considering real scenarios, with a size distribution of particles within surgical smoke, we carried out a simulation for a polydisperse medium [42], and assumed that the particle size followed a Gaussian distribution, with a range set to match surgical smoke particle sizes. The detailed simulation parameters are listed in Table 2. The distribution of particle sizes and the radiance of orthogonal linear polarization channels are shown in Fig. 3. We could conclude that, the particles polydispersity affects the contrast of orthogonal linear polarization channels, and the degree of impact was determined by the particle size distribution and weight. However, the co-polarized component still dominated the backscattering radiance.

**Table 2.** Simulation parameters

Parameter	Value
Mean diameter [34,35]	3.0 µm
Coefficient of variation of the Gaussian distribution [34,35,42]	0.3
Number of sampled points in the sphere size distribution [42]	1001
Illumination	Horizontal Linear Polarized
Wavelength	0.630 µm
Anisotropy *g*	0.7253
Scattering coefficient	0.7282 cm^−1^
Absorption coefficient [38,39]	0.0100 cm^−1^
Refractive index (Smoke particle) [40]	1.57 + 0.4277i
Refractive index (Tissue) [41]	1.50
Number of photons	50000
Depth of smoke layer	2 cm, 6 cm

From the simulation results we can conclude that, for the linear polarization imaging:

i) the co-polarized channels consist of large amounts of polarization-maintaining light from the superficial layers;
ii) the cross-polarized channels eliminate dominance of radiance from superficial layers, which means the cross-polarized channel has a certain dehazing effect, and this is consistent with the conclusions in Ref. [23,25];
iii) at greater depths, co-polarized and cross-polarized channels have similar intensities.

### 2.4 Polarization difference for transmission estimation

For linearly polarized incident light and a pair of orthogonal (co-polarized and cross-polarized) backscattered channels, the polarization difference calculation can be used to estimate parameters of the degradation model. As was demonstrated in the previous section and summarized in Table 3, the co-polarized channel was dominated by a polarization-maintaining component from smoke superficial layers [28]. After multiple particle interactions during propagation into deeper layers, the light had near-equal contribution in all polarization channels (including the co-polarized and the cross-polarized channels) [43]. The cross-polarized channels reduced the radiance from superficial layers and included a multiply scattered component. Therefore, compared with co-polarized channels, cross-polarized channels contained relatively more radiance from deeper smoke layers closer to the object.

**Table 3.** Backscattered light characteristics for linear polarization illumination

Illumination	Detection	Light component
Linear	Linear (co-polarized)	Polarization-maintaining photons + multiply-scattered photons
Linear	Orthogonal (cross-polarized)	Multiply-scattered photons

The polarization difference calculation was implemented to estimate the smoke component and transmission rate of the medium. The polarization-based image degradation model for co-polarized and cross-polarized states was rewritten as:

(5) $$\{\begin{array}{l} I_{co}\text{(x)}=J_{co}\text{(x)}e^{-\beta \tau (x)}+{\text{A}}_{co}(\infty )(1-e^{-\beta \tau (x)})\\ I_{c\text{r}o}\text{(x)}=J_{cro}\text{(x)}e^{-\beta \tau (x)}+{\text{A}}_{cro}(\infty )(1-e^{-\beta \tau (x)}) \end{array},$$

When the incident light underwent multiple scattering to reach the object layer, it lost its initial polarization state and became randomly polarized before being detected, therefore, the radiance from the object, $J_{co}\text{(x)}$ and $J_{cro}\text{(x)}$ could be regarded as approximately equal (when the detection distance approached the photon transport mean free path in the medium) [43] in Eq. (5). So that polarization difference calculation could be written as:

(6) $$I_{co}\text{(x)}-I_{c\text{r}o}\text{(x)}={\text{A}}_{co}(\infty )(1-e^{-\beta \tau (x)}),$$

Notably, ${\text{A}}_{co}(\infty )$ was a constant and was estimated from all the pixels of co-polarized channel, in the presence of complete smoke component. Therefore, ${\text{A}}_{co}(\infty )$ should be maintained in the polarization difference calculation. Then the transmission rate of the degradation model could be estimated as:

(7) $$t\text{(x)}=e^{-\beta \tau (x)}=1-\frac{I_{co}\text{(x)}-I_{c\text{r}o}\text{(x)}}{{\text{A}}_{co}(\infty )},$$

A smoke removal result was reconstructed by doubling the radiance of the processed cross-polarized channel because:

i) cross-polarized channels were less affected by the polarization-maintaining light as well as the reflectance of the medium [23,25];
ii) cross-polarized channels contained relatively more detailed information of the object (an advantage of polarization-resolved imaging);
iii) in deeper layers, the contribution of co-polarized and cross-polarized channels was nearly equal.

In summary, the restored image could be calculated as:

(8) $$J\text{(x)}=2\cdot J_{cro}\text{(x)}=2\cdot \frac{I_{c\text{r}o}\text{(x)}-{\text{A}}_{cro}(\infty )}{e^{-\beta \tau (x)}}+\text{2}\cdot {\text{A}}_{cro}(\infty ),$$

Note that even if the smoke is inhomogeneous, the difference of pixels under different optical depths is still reflected in the polarimetric imaging results and polarization difference results. The processing of each pixel is entirely independent and the estimated transmission rate *t* will vary corresponding to the smoke density, so our model can handle inhomogeneous smoke.

### 2.5 Estimation of ambient light with infinite optical depth

The estimation of ${\text{A}}_{co}(\infty )$ and ${\text{A}}_{cro}(\infty )$ can be contrasted with outdoor haze imaging methods, where a pixel is selected from a background area containing no visible object, representing an infinite depth point, i.e. an estimate of the air-light at infinity. However, in biomedical imaging where the tissue is generally close to the camera, there may be no areas with infinite optical depth to estimate the ambient light at infinity. Therefore, the algorithm in [44] was adopted to estimate the parameter A(∞) .

In brief, a specific color at different positions within the image will be affected by haze to varying degrees, and its red-green-blue radiance constitutes a linear distribution called a ‘Haze Line’. Pixels in a hazy image can be modeled using these haze-line priors in red-green-blue space that all intersect with the air-light value at infinity [44].

The method contains three main steps [44]:

i) converting the RGB image into an indexed image with 64 clusters (64 typical color values);
ii) projecting the color clusters onto three different 2D RGB planes, RB, GB, RG;
iii) using a Hough transform to vote for the location of the air-light in 2D RGB planes.

### 2.6 Color information

Color is affected by haze to varying degrees, and it is important to estimate the transmission rate *t* per pixel in R, G, B channels respectively, instead of using a single unique value per pixel for three channels. For example, if haze-free red pixels are affected by smoke, the intensities of the G and B channels are more significantly affected compared to the R channel and therefore, the transmission rate for the G and B channels must be lower than for the R channel [45].

## 3. Experiments

### 3.1 Experiment setup

**Fig. 4.**
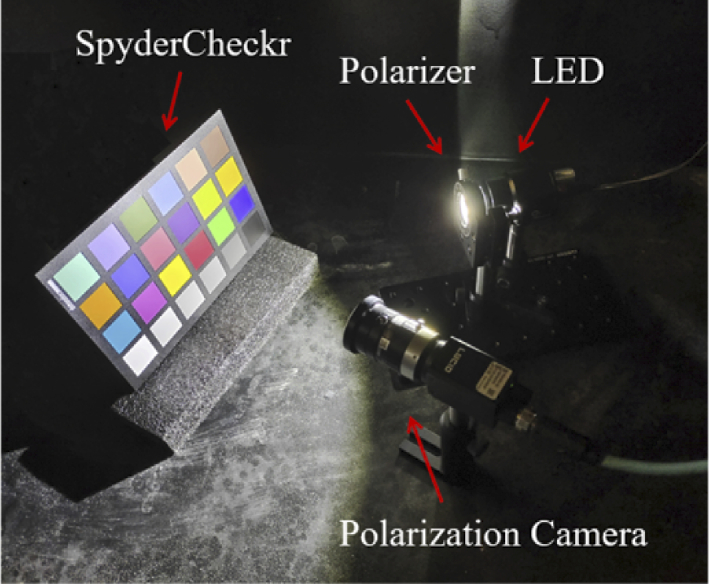
The polarization setup

The Polarization State Analyzer (PSA) for the experiments was a LUCID-TRI050S integrated with Sony polarized sensor IMX250MYR. With an intuitive graphical user interface, (Arenaview), four polarimetric channels, 0°, 45°, 90°, 135°, could be captured in color simultaneously. In addition, a linear polarization luminant was used as the polarization state generator (PSG) and consisted of a LED light source GI-0604 and a calibrated linear polarizer. The polarization setup is shown in Fig. 4. All the equipment was placed in a container with a black foam cover, and a fogger AB-900 could inject a controllable level of fog (from heated fluid) into the container through a pipe to simulate the surgical smoke environment. The created smoke contained multi-size particles and had a diameter mainly ranging from 1-5 µm [46]. A series of polarimetric images that varied with the concentration of the smoke were collected.

### 3.2 Results and analysis

**Fig. 5.**
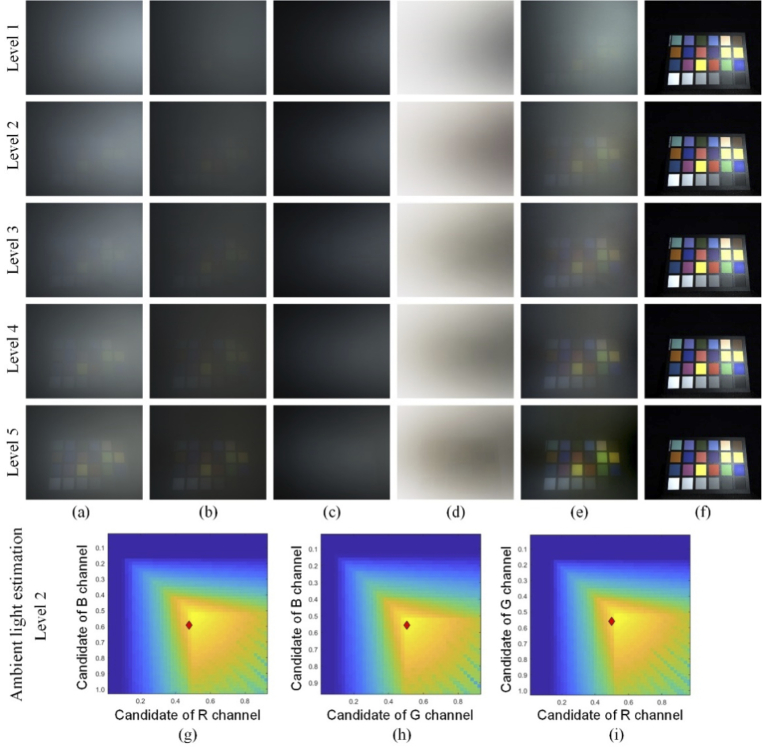
Comparison of different smoke concentrations (levels 1–5, where level 1 was extremely heavy smoke, level 3 was moderate and level 5 was mild concentration) with SpyderCheckr: (a) co-polarized smoke images, (b) cross-polarized smoke images, (c) polarization difference images, (d) estimated transmission maps, (e) smoke removal output images, (f) ground truth. (g-i) Examples of ambient light estimation with infinite optical depth (diamond) for level 2 smoke in different indicated color spaces.

**Fig. 6.**
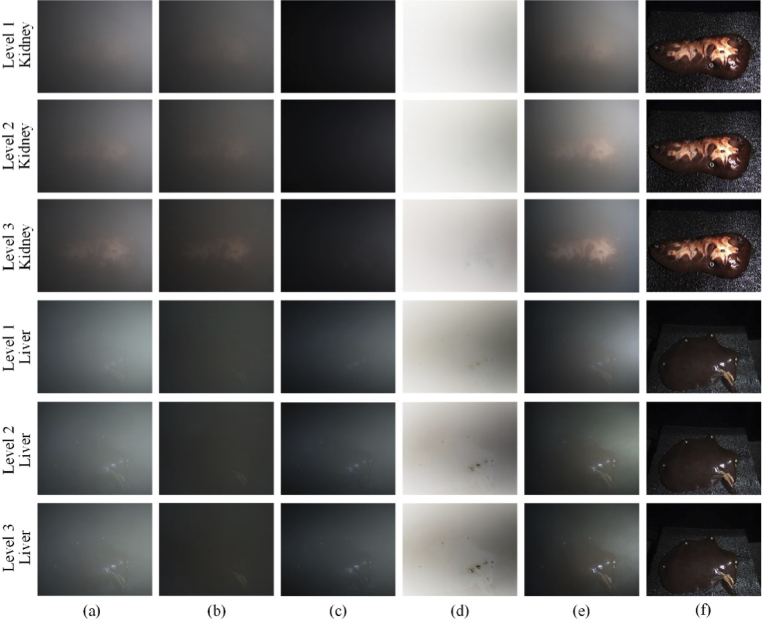
Comparison between different smoke concentrations for tissue samples (kidney and liver): (a) co-polarized smoke images, (b) cross-polarized smoke images, (c) polarization difference results, (d) estimated transmission maps, (e) smoke removal output images, (f) ground truth.

Before smoke injection, the incident linear polarized light was calibrated to the sample and smoke-free ground truth reference data was captured. Since the sample signal was completely obscured by the smoke, the polarimetric images of the mixed medium were collected at regular intervals (different smoke concentrations). A comparative experiment with a SpyderCheckr sample for different smoke concentrations is shown in Fig. 5, including examples of estimating the ambient light with infinite optical depth (Fig. 5(g-i)). A pig kidney and liver were chosen as *ex vivo* tissue samples, as shown in Fig. 6. A pair of orthogonal polarimetric images with 1134 × 917 pixels took an average processing time of 5.5 seconds using MATLAB R2019a on a Windows laptop with Intel Core i7-9750H and 16GB RAM. The experimental results show that the polarization-based smoke removal method reduced the effect of smoke on image quality in near real time for different smoke concentrations and samples.

**Fig. 7.**
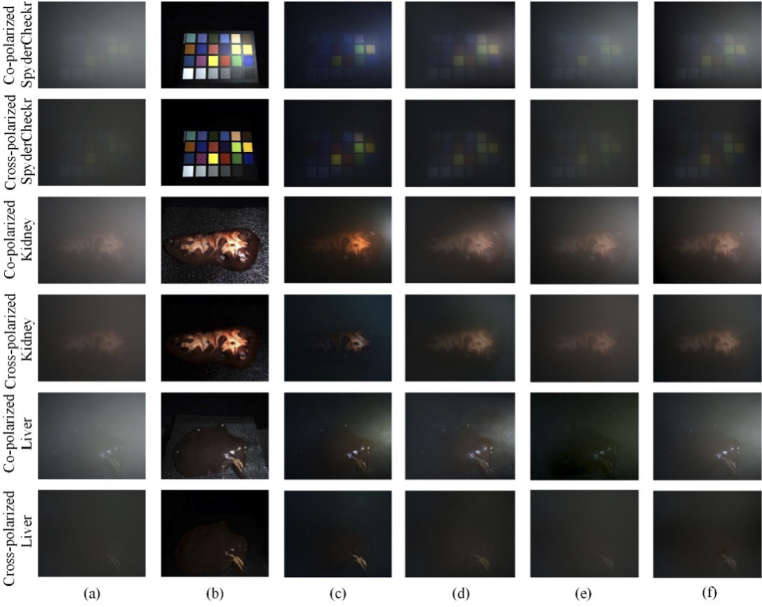
Comparison of dehazing results between different methods: (a) haze images, (b) ground truth, results processed by (c) DCP, (d) HLP, (e) CAP, (f) POL.

Three prior-based dehazing methods were also implemented for comparison, including methods based on a dark channel prior (DCP) [15], haze line prior (HLP) [19] and color attenuation prior (CAP) [20], with different sample surfaces. Both the co-polarized and cross-polarized images were processed by different dehazing methods, as shown in Fig. 7. According to our tests, these methods have similar processing times.

It can be concluded that all methods can reduce the impact of smoke on image quality. After comparing the dehazing result with the ground truth, we conclude that DCP method underestimate the transmission parameters, leading to a very low luminance, oversaturation effect and information loss, as shown in Fig. 7(c). The HLP method had a better dehazing performance, as indicated by Fig. 7(d), but compared to the ground truth, the restoration of the original color was not accurate. The CAP method overestimated the transmission parameters so that the smoke was not completely removed, and for the liver sample it was not accurate enough to restore the color, as shown in Fig. 7(e). Our polarization-based method (POL) reduced the impact of smoke, as indicated in Fig. 7(f), and through the comparison of the SpyderCheckr scenario, the original color was restored very well.

The Peak Signal-to-Noise Ratio (PSNR) [47], the Structural Similarity Index (SSIM) [48], the CIEDE2000 [49] were adopted to quantitatively evaluate the dehazing results, as shown in Table 4, for reference. The PSNR index was defined by Mean Square Error [47] and the SSIM index was determined by three factors, luminance, contrast and structure [48]; the higher the values of these two indicators, the better the image quality. The CIEDE2000 could evaluate color difference of dehazing results and ground truth [49], for color restoration performance, the lower the value of this indicator, the better the color restoration performance. We manually selected the pixels of the sample area and performed quantitative evaluation so as to reduce interference from the background. From these values we concluded that different methods usually have different performance in different scenarios. However, sometimes the conclusion from qualitative and quantitative analysis were not consistent, for example, we qualitatively considered that CAP did not have a good smoke removal performance on the co-polarized channel of the SpyderCheckr and liver sample because the parameters were overestimated and the color restoration was inaccurate, as shown in Fig. 7(e), but it had the best PSNR and SSIM results.

**Table 4.** Quantitative evaluation of different dehazing methods: SSIM and PSNR (the higher the better), CIEDE2000 (the lower the better)

**Surface Type & Channel**	**Index**	**DCP**	**HLP**	**CAP**	**POL**
	SSIM	0.9804	0.9855	0.9891	0.9882
**SpyderCheckr Co**	PSNR	56.6853	57.8997	59.0408	58.7155
	CIEDE2000	0.1115	0.0951	0.1018	0.0950
	SSIM	0.9933	0.9948	0.9941	0.9942
**Kidney Co**	PSNR	61.3353	62.2904	61.7940	61.8730
	CIEDE2000	0.1153	0.1153	0.1155	0.1151
	SSIM	0.9981	0.9974	0.9985	0.9958
**Liver Co**	PSNR	66.5942	65.3429	67.4119	63.4221
	CIEDE2000	0.0137	0.0137	0.0137	0.0137

**Fig. 8.**
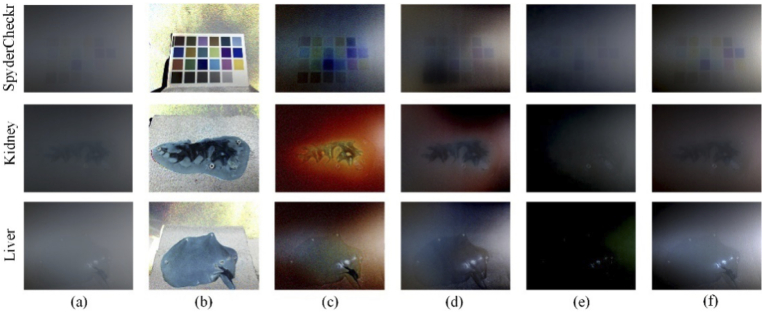
Comparison of restored DOP results between different methods: (a) the DOP of haze images, (b) the DOP of ground truth, results processed by (c) DCP, (d) HLP, (e) CAP, (f) POL.

However, in addition to restoring the image visibility, for biomedical images, the restoration of the original polarization properties of the samples can be further applied for biomedical information processing and disease detection. Therefore, it may be useful to restore the original polarization information of the samples as far as possible while reducing the influence of surgical smoke. Muller matrices can characterize the sample’s polarization properties and evaluate the accuracy of polarization information restoration. However, it needs multiple measurements to obtain and is not suitable for dynamic smoke scenarios. Therefore, the degree of polarization (DOP), one of a number of possible parameters describing the polarization information of the samples, was adopted and calculated using Eq. (9). The restored DOP results are shown in Fig. 8 and quantified in Table 5. In the SpyderCheckr scenario, the DCP and HLP method were not accurate enough for color recovery, which would affect the balance between polarization channels and therefore affect the DOP restoration. The CAP method had limited ability to recover DOP information. Compared with the qualitative and quantitative results, our POL-based method had the best DOP restoration performance.

(9) $$\text{DOP}=\frac{I_{co}\text{(x)}-I_{c\text{r}o}\text{(x)}}{I_{co}\text{(x)}+I_{c\text{r}o}\text{(x)}},$$

However, our method also has shortcomings, since cross-polarized radiance can inevitably be affected by smoke, especially at high concentrations where the estimated smoke information from polarization difference calculations is lower than expected, which in turn leads to overestimation of the transmission parameters. Therefore, in the case of the environment with high smoke concentration, our experimental results still retained some smoke. On the other hand, in real surgical smoke scenarios, the concentration of smoke would not be expected to reach that level [1], so the proposed polarization-based smoke removal method remains applicable. In addition, the proposed quantitative evaluation indexes often evaluate from a certain aspect, and cannot comprehensively evaluate the smoke removal performance, so they are not entirely suitable for our scenario. Specifically, PSNR only considers the absolute difference and is not sensitive to structural information; SSIM is partially determined by a luminance factor, however, in our surgical smoke scenario, the captured smoke images have higher luminance than processed images, so they have higher scores in luminance factors than processed images. This is confounding because the processed images should have a higher SSIM score than the smoke images, and it is therefore important to better balance the weights of these three SSIM factors, or to develop other quantitative evaluation indexes for our scenario.

**Table 5.** Quantitative evaluation of DOP results of different methods: SSIM and PSNR (the higher the better), CIEDE2000 (the lower the better)

**Surface Type**	**Index**	**DCP**	**HLP**	**CAP**	**POL**
	SSIM	0.9810	0.9812	0.9819	0.9864
**SpyderCheckr**	PSNR	56.3261	56.4478	56.5600	57.7247
	CIEDE2000	0.2244	0.2040	0.1962	0.1949
	SSIM	0.9641	0.9649	0.9555	0.9661
**Kidney**	PSNR	53.9179	54.0238	53.0454	54.1689
	CIEDE2000	0.3771	0.1636	0.1635	0.1635
	SSIM	0.9654	0.9707	0.9343	0.9758
**Liver**	PSNR	54.0115	54.7181	51.3724	55.5464
	CIEDE2000	0.2821	0.2469	0.2389	0.2384

## 4. Conclusion

In endoscopic/laparoscopic surgery, image quality can be severely degraded by surgical smoke, which reduces the visibility for the surgeons and introduces errors when using image processing. A simple, real-time, polarization-based smoke removal method for surgical images was proposed. Unlike the outdoor dehazing problem, we redefined the parameters of transmission rate and ambient light with infinity and proposed the polarization-based degradation imaging model. Experiments with a calibrated polarization setup, for different smoke concentrations, and different sample surface materials were conducted. Qualitative and quantitative analysis and comparisons with alternative methods showed that our method can effectively reduce the degradation of biomedical images caused by surgical smoke and partially restore the original degree of polarization of the samples.

## Data Availability

Data underlying the results presented in this paper are not publicly available at this time but may be obtained from the authors upon reasonable request.

## Supplemental document

See Supplement 1 for supporting content.
