# Supplementary material for: Polarization-based smoke removal method for surgical images
Source: Biomed Opt Express. 2022 Mar 22;13(4):2364–79. doi: 10.1364/BOE.451517 (PMC9045924; doi:10.1364/BOE.451517)
Supplement: Supplementary file 1 [file boe-13-4-2364-s001.pdf]

Supplemental Document

Biomedical Optics EXPRESS

Polarization-based smoke removal method for surgical images: supplement

DAQIAN WANG,[1,2,3,6] JI QI,[4] BAORU HUANG,[2,3] ELIZABETH NOBLE,[2,3] DANAIL STOYANOV,[5] JUN GAO,[1] AND DANIEL S. ELSON[2,3,7]

[1] *School of Computer and Information, Hefei University of Technology, Hefei, 230601, China*
[2] *Hamlyn Centre for Robotic Surgery, Imperial College London, London, SW7 2AZ, UK*
[3] *Department of Surgery and Cancer, Imperial College London, London, SW7 2AZ, UK*
[4] *Research Center for Intelligent Sensing, Zhejiang Lab, Hangzhou, 311100, China*
[5] *Department of Computer Science, University College London, London, WC1E 6BT, UK*
[6] *dw619@mail.hfut.edu.cn*
[7] *ds.elson@imperial.ac.uk*

This supplement published with Optica Publishing Group on 22 March 2022 by The Authors under the terms of the Creative Commons Attribution 4.0 License in the format provided by the authors and unedited. Further distribution of this work must maintain attribution to the author(s) and the published article's title, journal citation, and DOI.

Supplement DOI: https://doi.org/10.6084/m9.figshare.19355462

Parent Article DOI: https://doi.org/10.1364/BOE.451517

A polarization-based smoke removal method for surgical images: supplemental document

The purpose of the modeling was to study the propagation of polarized photons interacting with the environment containing the medium and the object, in order to provide theoretical guidance for the measurement experiments. The modeling was based on the Monte Carlo statistical method, by tracking the photons emission process, scattering, reflection and update of the corresponding polarization state in the environment, and then counting the energy of polarized photons received by the detector. The model flow chart is shown in Fig. S1.

Ref. [1,2] provide a polarimetric scattering modeling method, including an update of the photon movement state and polarization state after scattering. Please refer to the source code for details:

https://omlc.org/software/polarization/

Ref. [3,4] provide a geometric modeling method of rough surfaces, including the determination of a photon's reflection direction. Please refer to the source code for details:

https://tatsy.github.io/blog/applications/graphics/1742/
https://github.com/tatsy/MicrofacetDistribution

Ref. [5-9] provide a polarimetric reflection modeling method, including the determination of a reflected photon's Mueller Matrix and updated of polarization state. Please refer to the source code for details:

https://pages.nist.gov/SCATMECH/docs/index.htm

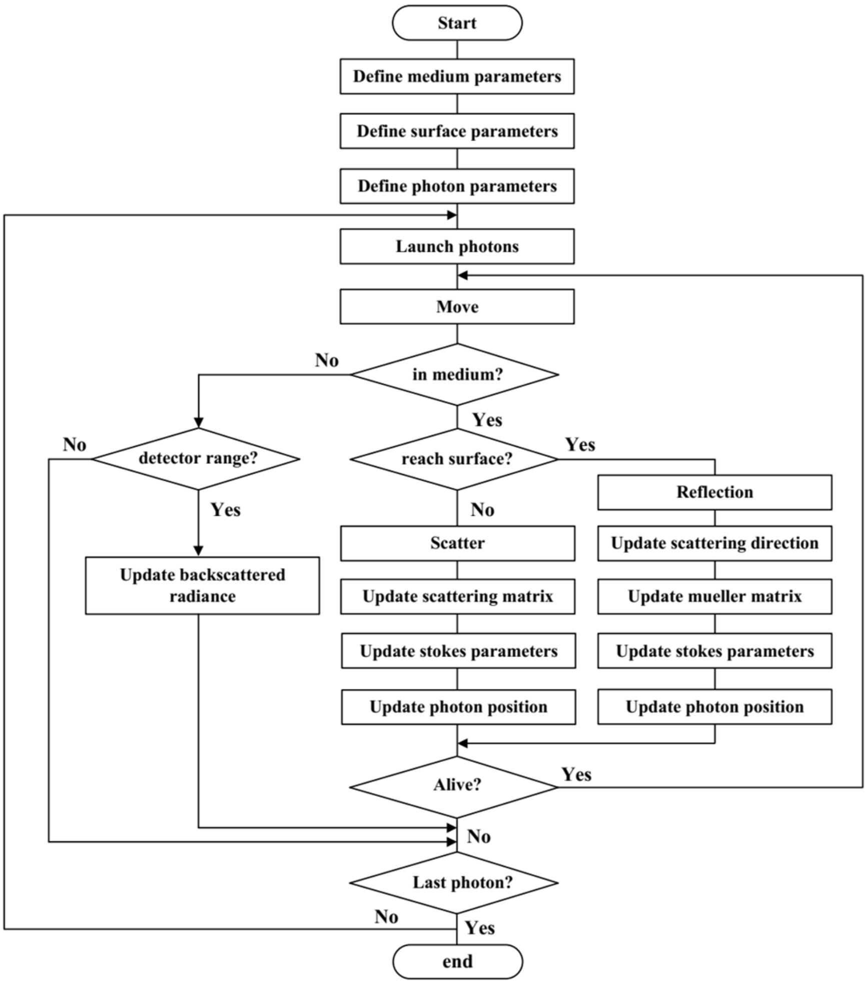

Fig. S1 The modeling method flow chart

References

1. J. C. Ramella-Roman, S. A. Prahl, and S. L. Jacques, “Three Monte Carlo programs of polarized light transport into scattering media: part I,” Optics Express **13**(12), 4420-4438 (2005).
2. J. C. Ramella-Roman, S. A. Prahl, and S. L. Jacques, “Three Monte Carlo programs of polarized light transport into scattering media: part II,” Optics express **13**(25), 10392-10405 (2005).
3. B. Walter, S. R. Marschner, H. Li, and K. E. Torrance, “Microfacet Models for Refraction through Rough Surfaces,” Rendering techniques, 2007, 18th (2007).
4. E. Heitz, “Understanding the masking-shadowing function in microfacet-based BRDFs,” Journal of Computer Graphics Techniques **3**(2), 32-91 (2014).
5. R. G. Priest, and T. A. Gerner, “Polarimetric BRDF in the microfacet model: Theory and measurements,” NAVAL RESEARCH LAB WASHINGTON DC (2000).
6. T. A. Germer, “Angular dependence and polarization of out-of-plane optical scattering from particulate contamination, subsurface defects, and surface microroughness,” Applied Optics **36**(33), 8798-8805 (1997).
7. T. A. Germer, C. C. Asmail, and B. W. Scheer, “Polarization of out-of-plane scattering from microrough silicon,” Optics letters **22**(17), 1284-1286 (1997).
8. T. A. Germer, and Egon Marx, “Ray model of light scattering by flake pigments or rough surfaces with smooth transparent coatings,” Applied optics **43**(6), 1266-1274 (2004).
9. T. A. Germer, "SCATMECH: polarized light scattering C++ class library." (2000).
